# New crystal forms and amorphous phase of sophoricoside: X-ray structures and characterization

**DOI:** 10.1098/rsos.181905

**Published:** 2019-01-23

**Authors:** Cheng Xing, Guo shun Zhang, Ning bo Gong, Guan hua Du, Yang Lu

**Affiliations:** 1 Beijing Key Laboratory of Polymorphic Drugs, Chinese Academy of Medical Sciences and Peking Union Medical College, Beijing 100050, People's Republic of China; 2 Beijing City Key Laboratory of Drug Target Identification and Drug Screening, Institute of Materia Medica, Chinese Academy of Medical Sciences and Peking Union Medical College, Beijing 100050, People's Republic of China

**Keywords:** sophoricoside, solvatomorphism, crystal structure, amorphous, solubility

## Abstract

Sophoricoside, which is an isoflavone glycoside found in many plant species, has recently attracted attention because of its anti-fertility activity. One solvent-free form, two solvatomorphs and an amorphous phase of sophoricoside are reported for the first time. X-ray diffractometry, differential scanning calorimetry, thermal gravimetric analysis and Fourier-transform infrared spectroscopy were used to characterize the different forms. The results show that factors such as crystal symmetry, intermolecular arrangement, conformational flexibility, hydrogen-bonding interactions and solvent incorporation lead to different solid-state forms. An investigation of the transformations of the four forms showed that they can interconvert with each other under certain conditions. Amorphous phase and solvatomorphism were unstable but can improve the solubility of sophoricoside in water.

## 1. Introduction

Polymorphs are substances with different arrangements and/or conformations in the crystal lattice that consist of the same elemental composition, including crystalline state and amorphous state [1]. Generally, with respect to crystalline state, the stability of amorphous state is poor and the solubility of amorphous state is good. Solvatomorphism is defined as a substance to form different unit cells, where these unit cells vary in their elemental compositions through the inclusion of one or more solvent molecules [2]. Different solid-state forms of an active pharmaceutical ingredient can exhibit significant diversity in physicochemical and biological properties, e.g. density, fluidity, melting point, solubility, dissolution rate, stability, membrane permeability and bioavailability [3]. Although the existence of the organic solvents in therapeutic substance may significantly raise the toxicity of solvatomorphism, it could still be of great valuable for its research potential. For example, organic solvated forms of some drugs are final products for clinical use. Two Food and Drug Administration (FDA)-approved drugs (cabazitaxel and trametinib) were in the form of solvates with acetone and dimethyl sulfoxide (DMSO) incorporated, respectively [4,5]. Moreover, the significance of solvatomorphism is also reflected in their potential contributions of new polymorphic forms obtained through the removal of solvent, the convenience of available single-crystal X-ray diffraction (SXRD) data and their purification and patent aspect [6–10].

**Figure 1.**
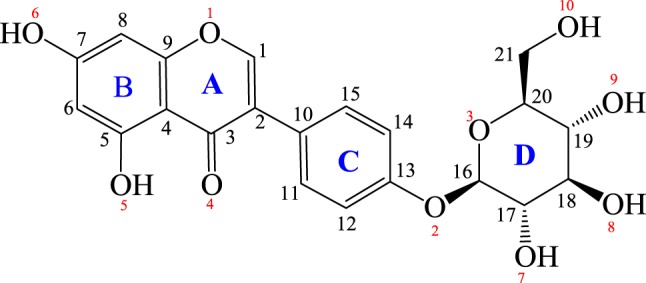
Chemical structure of sophoricoside.

Sophoricoside (C_21_H_20_O_10_), which has the molecular structure shown in figure 1, is an isoflavone glycoside; its chemical name is 5,7-dihydroxy-3-(4-(((2R,3S,4R,5R,6S)-3,4,5-trihydroxy-6-(hydroxymethyl)tetrahydro-2H-pyran-2-yl)oxy)phenyl)-4H-chromen-4-one. Sophoricoside is mainly present in the dry flowers, buds and ripe fruits of *Sophora japonica* L., and can also be extracted and separated from *Ginkgo biloba* L [11]. Pharmacological studies have shown that sophoricoside has anti-inflammatory [12] and anti-fertility activities [13,14]; it is effective in the prevention and treatment of osteoporosis in postmenopausal women [15], promotes osteogenesis and inhibits bone loss [16]. Previously reported studies of sophoricoside have concentrated on areas such as extraction, derivatives, pharmacology and content determination, but systematic studies of crystal structures and solid-state forms have not been reported [17,18].

In this study, four solid-state forms (A, B, C and D) of sophoricoside were discovered; form A is solvent free, form B incorporates dimethyl sulfoxide (DMSO), form C incorporates pyridine and water and form D is amorphous phase. The four forms of sophoricoside were characterized using single-crystal X-ray diffraction (SXRD), powder X-ray diffraction (PXRD), differential scanning calorimetry (DSC), thermogravimetric analysis (TGA) and Fourier-transform infrared (FT-IR) spectroscopy. The main factors leading to four forms of sophoricoside are discussed. The stability and solubility of four forms were investigated.

## 2. Material and methods

### 2.1. Materials

Sophoricoside was purchased from the Hubei Ju Sheng Trade Co., Ltd (Hubei Province, China, batch number: 130616). The chemical purity, which was determined using high-performance liquid chromatography [19], was higher than 99.0%. All solvents used were analytical reagent grade.

### 2.2. Sample preparation and crystallization

Form A was obtained by completely dissolving sophoricoside in a mixture of acetone and water (1 : 1, v/v). The saturated solutions were allowed to stand for crystallization at 25°C for about 10 days.

Form B was prepared by completely dissolving sophoricoside in a mixture of DMSO and tetrahydrofuran (1 : 10, v/v). The saturated solutions were allowed to stand for crystallization at 10°C for about 5 days.

Form C was prepared by completely dissolving sophoricoside in a mixture of pyridine and water (20 : 1, v/v). The saturated solutions were allowed to stand for crystallization at 25°C for about 12 days.

Form D was obtained by physical mechanical ball mill for 5 h. The mass ratio of agate ball to sophoricoside is 6 : 1 and ball mill machine speed is 400 r.p.m.

### 2.3. X-ray crystallography

SXRD data for the A, B, C forms of sophoricoside were obtained using a MicroMax 002+ diffractometer equipped with a Cu fine-focus sealed tube and a 0.3 mm MonoCap collimator. The structures were solved by direct methods, using SHELXS-97, and refined by full-matrix least-squares refinement on *F*^2^ with anisotropic displacement parameters for non-hydrogen atoms, using SHELXL-97. All hydrogen atoms were refined isotropically and were placed in calculated positions using riding models.

PXRD analysis of the A, B, C and D forms of sophoricoside was performed using a D/max-2550 (Rigaku, Japan) X-ray diffractometer with graphite monochromatized Cu Kα (*λ* = 1.54187 Å) radiation at room temperature. Powders for PXRD were obtained by grinding crystalline materials in an agate mortar to a particle size of around 5 µm. The data were recorded in the angular range 3°–40° (2*θ*) with a step size of 0.02° and a scanning speed of 8° min^−1^. Simulated powder patterns of A, B and C forms were obtained from the single-crystal data using Mercury 2.4.

### 2.4. Thermal analysis

DSC and TGA were performed with a Mettler-Toledo TGA/DSC STARe system (Mettler, Switzerland) under an atmosphere of dry N_2_ flowing at 50 cm^3^ min^−1^. DSC was performed in the range 30–300°C at a rate of 10°C min^−1^; TGA was performed in the range 30–500°C at a rate of 10°C min^−1^. The TGA/DSC data were analysed using STARe software.

### 2.5. FT-IR spectroscopy

FT-IR absorption spectra were recorded with a Spectrum 400 FT-IR spectrophotometer (PerkinElmer, USA). The spectra were used to identify the functional groups in the four forms of sophoricoside. The spectra were collected in the range 650–4000 cm^−1^ with a 4 cm^−1^ resolution. An attenuated total reflectance sampling accessory with a diamond window was used.

### 2.6. Solid-state milling

Form D of sophoricoside was obtained by using a Pulverisette 6 ball mill (Fritsch, German), using 250 ml agate containers with 20 agate ball (Ø 15 mm), and ball mill machine speed is 400 r.p.m.

### 2.7. Solubility

Solubility experiments were conducted on a constant-temperature ZWY-103B oscillator (Zhicheng Analytical Instrument Manufacturing Co. Ltd, Shanghai, China). Excess amounts of four solid-state forms of sophoricoside were added to pure water (pH 7.0), which were heated to 310 K in advance and the solutions were shaken at 310 K for 4 h at 200 r.p.m. The suspensions were filtered through 0.2 mm membrane filters, and the filtrate was diluted and measured by Agilent1200 HPLC (Agilent Technologies, USA). The chromatograph conditions were as follows: the mobile phase, methanol and 0.1% phosphoric acid aqueous solution (v/v = 48 : 52); injection volume, 10 ml; detection wavelength, 260 nm; column temperature, 303 K; flow rate, 1.5 ml min^−1^.

## 3. Results

### 3.1. SXRD

The crystallization of a specific compound is governed by the compound's inherent nucleation and growth properties. During crystal growth, sophoricoside molecules dispersed in solvents with various properties (polarity, polarizability and hydrogen-bonding propensity) and interacted with the solvent molecules. Consequently, different nuclei can be assembled during desaturation. If the different nuclei are allowed to grow, different crystal forms are obtained [20,21]. Solvatomorphism formation also depends on the properties of the surrounding environment, such as temperature, humidity and the rate of generation of supersaturation, but the solvent has the most important impact [22].

The crystallographic data and refinement details for the A, B and C forms of sophoricoside are listed in table 1. This is the first time that crystallographic data of these sophoricoside crystal forms have been reported. The crystals of the three forms are all prisms, but they crystallize in different space groups. Form A is orthorhombic, with the chiral space group P2_1_2_1_2_1_; each asymmetric unit contains a sophoricoside molecule. Form B is monoclinic, with the chiral C2 space group; it consists of a sophoricoside molecule and 0.5 of a crystallized DMSO molecule. Form C is monoclinic, with the chiral P2_1_ space group; it consists of five sophoricoside molecules, 11 pyridine molecules and five water molecules in an asymmetric unit. Because of the disordered thermal motion, the solvent molecules of forms B and C have a higher temperature factor.

**Table 1.** Crystal parameters of A, B and C forms of sophoricoside.

compound	form A	form B	form C
colour/shape	colourless/prism	colourless/prism	colourless/prism
crystal size (mm)	0.02 × 0.12 × 0.35	0.08 × 0.40 × 0.67	0.17 × 0.23 × 0.57
empirical formula	C_21_H_20_O_10_	C_21_H_20_O_10_·(C_2_H_6_O_1_S)_0.5_	(C_21_H_20_O_10_)_5_(C_6_H_5_N_1_)_11_·(H_2_O)_5_
formula weight	432.37	471.45	3122.03
crystal system	orthorhombic	monoclinic	monoclinic
space group	P2_1_2_1_2_1_	C2	P2_1_
*a* (Å)	6.197(4)	43.030(30)	23.174(6)
*b* (Å)	7.710(3)	7.562(4)	13.823(2)
*c* (Å)	38.496(13)	6.403(4)	24.200(4)
*α* (°)	90.00	90.00	90.00
*β* (°)	90.00	91.72(3)	103.246(3)
*γ* (°)	90.00	90.00	90.00
volume (Å^3^)	1839(2)	2083(2)	7546(3)
*Z*	4	4	10
density (g cm^−3^)	1.561	1.504	1.374
*F* (000)	904	988	3284
theta range for data	4.59 < *θ* < 72.14	4.11 < *θ* < 65.99	3.75 < *θ* < 72.65
*h/k/l* ranges	−7 ≤ *h* ≤ 6	−50 ≤ *h* ≤ 50	−27 ≤ *h* ≤ 14
	−8 ≤ *k* ≤ 9	−8 ≤ *k* ≤ 8	−16 ≤ *k* ≤ 17
	−47 ≤ *l* ≤ 44	−3 ≤ *l* ≤ 7	−29 ≤ *l* ≤ 29
reflections collected	3419	3053	28 302
independent reflections	2132	2843	22 550
completeness (%)	96.5	98.4	97.6
final *R*, *wR* (*F*^2^) values [*I* > *2σ(I)*]	0.0925, 0.2125	0.0431, 0.1189	0.0871, 0.2239
final *R*, *wR*(*F*^2^) values (all)	0.1112, 0.2289	0.0449, 0.1213	0.1022, 0.2421
*R*_int_	0.1555	0.0504	0.1253
Flack	0.4(5)	0.05(5)	0.38(13)
GooF	1.008	1.001	1.087
temperature (K)	298	298	100
CCDC number	1812874	1812875	1812876

The crystal density value of form A was higher than those of forms B and C; this shows that the sophoricoside molecules of form A were arranged tightly in three-dimensional space. The calculated volume (227.8 Å^3^) of solvent-accessible voids per unit-cell volume in form B was 10.9%, but for form C the corresponding calculated volume was 2758.0 Å^3^, accounting for approximately 36.6% of the unit-cell volume. It is easy to see that the arrangement of all the molecules in form C was looser than that in form B. The Flack parameter of form B was refined as 0.05(5) and its absolute configuration was determined [23]. The Flack parameters of the other two forms were refined as 0.4(5) and 0.38(13), but their absolute configurations could not be properly determined. Moreover, the *R* (int) values of forms A and C are 0.15 and 0.12. *R* (int) is the *R*-value for averaging equivalent reflections and for small organic molecules such as those reported here would typically be 0.03–0.05. The unacceptably high Flack parameters and *R* (int) values of forms A and C are attributed to poor crystallographic data. In order to obtain better quality samples for re-measuring crystallographic data, the researchers cultured single crystal many times. But the crystals were too small, and the crystals were easily changed in the air for a long time. The best data are presented in this article.

**Figure 2.**
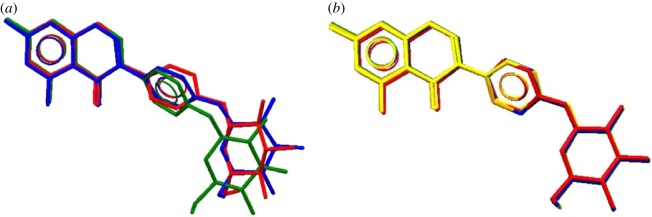
(*a*) Overlay of forms A–C (red, form A; blue, form B; green, form C); (*b*) overlay of five molecules of form C (red, molecule a; blue, molecule b; green, molecule c; orange, molecule d; and yellow, molecule e).

Rings A (O_1_, C_1_, C_2_, C_3_, C_4_, C_9_), B (C_4_, C_5_, C_6_, C_7_, C_8_, C_9_) and C (C_10_, C_11_, C_12_, C_13_, C_14_, C_15_) are planar, and ring D (O_3_, C_16_, C_17_, C_18_, C_19_, C_21_) adopts a chair conformation in all three forms, but the twist degrees of the rings are not the same. In form A, the C_1_–C_2_–C_10_–C_15_ torsion angle is −42.23° and the C_13_–O_2_–C_16_–O_3_ torsion angle is 78.13°. However, in forms B and C, the C_1_–C_2_–C_10_–C_15_ torsion angles are 38.91° and 38.57°, respectively, and the C_13_–O_2_–C_16_–O_3_ torsion angles are −78.07° and −74.74°, respectively. Correspondingly, the dihedral angles of different rings are dissimilar in the three forms. In figure 2*a*, form A (red), form B (blue) and one molecule of form C (green) are superimposed. The five host molecules of form C were overlaid in figure 2*b*. In the overlay diagrams, there are no clear conformational differences among the five molecules of form C, but conformational differences among the three forms arise because of the ring conformers and single-bond rotation.

**Figure 3.**
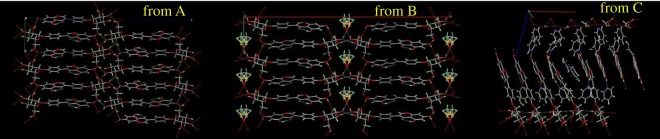
Schematic diagram of hydrogen bonds in A, B and C forms of sophoricoside.

In the three forms of sophoricoside, the carbonyl is linked to the hydrogen on the adjacent phenolic hydroxyl via intramolecular hydrogen bonds to form a cyclic structure. In the three structures, *π*–*π* stacking interactions between rings were identified and can be divided into two types, i.e. head-to-tail and head-to-head; the latter is rarer in flavonoid glycosides. The crystal structure of form A contains only sophoricoside molecules, which contain aromatic rings and a number of hydroxyl groups. The molecules mainly interact through *π*–*π* stacking and intermolecular hydrogen bonds. There are two phenolic hydroxyl groups on the benzene ring; because of their spatial proximity, the O_5_ atom acts as a proton donor and O_4_ acts as an acceptor to form an intramolecular hydrogen bond of length 2.611 Å. The O_6_ atom acts as both a proton acceptor and proton donor, alternately forming intermolecular hydrogen bonds with O_7_ and O_10_ of the glycosyl along the b-axis; intermolecular contacts involve head-to-tail *π*–*π* stacking interactions along the b-axis. The asymmetric unit in form B contains one sophoricoside molecule and 0.5 of a DMSO molecule. Form B mainly relies on intermolecular forces and *π*–*π* stacking interactions to maintain its three-dimensional structure. DMSO is involved in the formation of intramolecular and intermolecular hydrogen bonds. DMSO is located in the plane of symmetry and disordered; it occupies two positions, with shares of 0.5 and 0.5. The asymmetric unit in form C contains five sophoricoside molecules, five water molecules and 11 pyridine molecules. The O_5_ atom on the phenyl ring of the sophoricoside molecule, as in forms A and B, forms an intramolecular hydrogen bond with the carbonyl group, but the other phenolic hydroxyl group (O_6_) and the basic pyridine molecule are linked through an intermolecular hydrogen bond. The 11 pyridine molecules in the asymmetric unit can be divided into three classes: five pyridines form intermolecular hydrogen bonds with acidic phenolic hydroxyl groups; five pyridines form intermolecular hydrogen bonds with hydroxyl groups on the sophoricoside glycosyl group; and one pyridine interacts with the aromatic ring of a host molecule by *π*–*π* stacking interactions. Calculation results show that the distance between adjacent glycosyl group in sophoricoside is 4.5–4.7 Å. One pyridine ring is embedded in every five sophoricoside molecules, therefore the distance between the aromatic rings of sophoricoside is 3.9–4.0 Å; this indicates strong *π*–*π* stacking interactions, which can maintain the stability of the three-dimensional structure. The pyridine ring embedded in the aromatic ring position causes disorder, and occupies two positions, with shares of 0.5 and 0.5. Water molecules act as both hydrogen-bond acceptors and hydrogen-bond donors, connecting adjacent sophoricoside molecules via three hydrogen bonds. A comparison of the three crystal structures of sophoricoside shows that solvatomorphism is caused not only by the conformational flexibility of sophoricoside, but also the large number of hydroxyl groups in sophoricoside, which can form hydrogen bonds with some solvents. Schematic diagram of the hydrogen bonds involved in packing of the three forms of sophoricoside are shown in figure 3.

**Figure 4.**
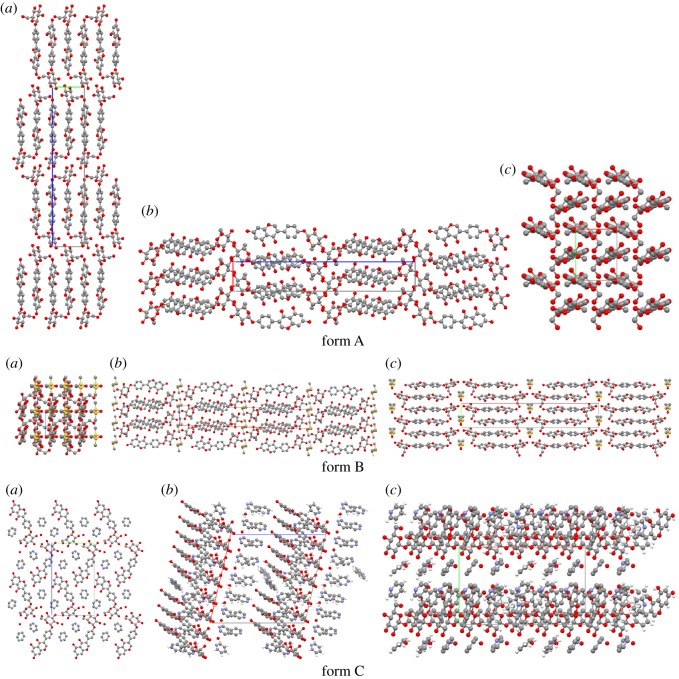
Molecular packing of A, B and C forms of sophoricoside viewed along crystallographic *a*-, *b*- and *c*-axes, respectively, from the left to right. Oxygen atoms are in red, carbon atoms are in grey, sulfur atoms are in yellow, and nitrogen atoms are in blue.

Figure 4 shows the differences among the molecular arrangements, viewed along the three axes in the A, B, C forms of sophoricoside. Form A forms an infinite chain via head-to-tail connections along the *c*-axis, and forms layers along the *b*-axis. Form B forms an infinite chain via tail-to-tail connections along the *a*-axis, with a DMSO molecule embedded in every two sophoricoside molecules, and forms layers along the *b*-axis. However, for form C, the most notable feature of the molecular arrangement is that the pyridine molecules are arranged in cavities formed by sophoricoside molecules, which results in a Z-chain structure when viewed along the crystallographic *a*-axis, and forms layers along the *b*-axis.

### 3.2. PXRD

**Figure 5.**
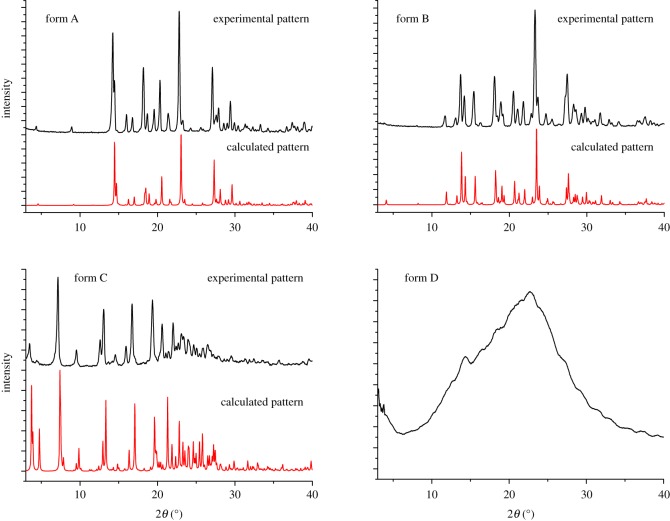
Experimental and calculated PXRD patterns for four forms of sophoricoside.

The SXRD data represent the imagination of one crystal, whereas PXRD data represent the imagination of many small crystals. The calculated PXRD pattern can, therefore, be regarded as the standard map of a crystal with 100% crystalline purity. The consistency of the SXRD and PXRD pattern can reveal the crystalline purity of the powder sample [24]. To confirm that the samples were the pure crystal forms, the PXRD patterns were recorded and compared with the theoretical powder patterns; good consistency was observed. The PXRD patterns of the four forms of sophoricoside show significant differences, therefore, the different forms can be easily distinguished. The experimental and calculated powder patterns of the four forms are shown in figure 5. The positions and relative intensities of the 10 most intense peaks are listed in electronic supplementary material, table S1.

### 3.3. Thermal analysis methods

**Figure 6.**
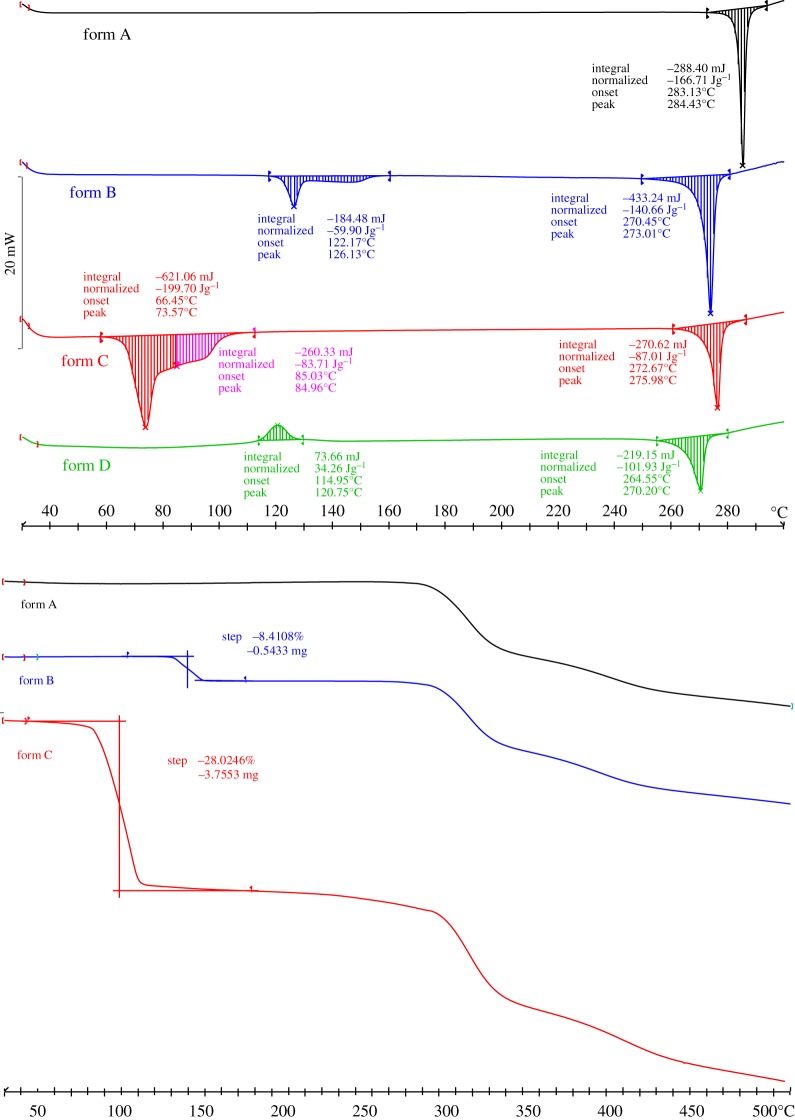
DSC thermograms and TGA curves for sophoricoside forms.

Thermal analysis methods can be used to clearly distinguish among four solid-state forms. Figure 6 shows the DSC profiles of the solid-state forms of sophoricoside. The DSC profile of form A shows a single endothermic peak at 284.43°C; this is ascribed to the melting point of sophoricoside; no solvent is present. For forms B and C, the DSC traces show an initial endothermic peak (126.13°C, form B; 73.57 and 84.96°C, form C), and a second endothermic peak (273.01°C, form B; 275.98°C, form C). This indicates that both forms B and C give a desolvation endothermic peak before the melting endothermic peak. The DSC thermograms indicate that solid-state form transformations of forms B and C to form A could occur during heating. This is verified by the PXRD patterns of form B after heating at 160°C for 10 min, and form C after heating at 100°C for 10 min. For form D, the DSC traces show a small exothermic peak (120.75°C) and an endothermic peak (270.20°C). It indicated that the exothermic peak (120.75°C) is the turning point of form D. Form D could transform to form A during heating. This is verified by the XPRD patterns of form D after heating at 120°C for 30 min (electronic supplementary material, figure S1).

The solvate stoichiometry was determined by measuring the mass loss in certain temperature ranges, using a thermal gravimetric analyser (figure 6). Form A shows only the melting decomposition peaks of sophoricoside. Forms B and C show both the exothermic peak of the solvate and melting decomposition peak of sophoricoside. The determined mass losses (w/w) for forms B and C are 8.0% and 27.5%, respectively.

The solvate stoichiometry was calculated as $n\times M_{\mathrm{solvent}}/(n\times M_{\mathrm{solvent}}+M_{\mathrm{sample}})=R$ [25]. Where *n* is the solvate stoichiometry; *M*_solvent_ and *M*_sample_ are the molar mass of the solvent and sample; and *R* is the solvate mass loss. The DMSO stoichiometry in form B is 0.48; the pyridine/water stoichiometry in form C is 1.91/0.87, which is in agreement with the SXRD results.

### 3.4. FT-IR spectroscopy

**Figure 7.**
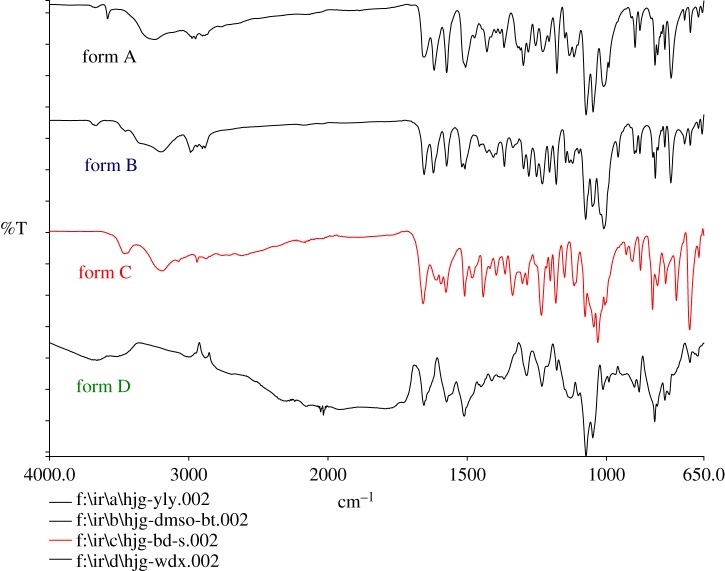
FT-IR spectra of sophoricoside forms.

All the sophoricoside forms were easily identified by their FT-IR spectra, which are shown in figure 7. Sophoricoside contains alcoholic hydroxyl and phenolic hydroxyl groups. The bands in the 3600–3100 cm^−1^ region are attributed to the O–H stretching vibrations of hydrogen-bonded alcohols and phenols. The band at 3463 cm^−1^ is attributed to crystallization water in form C. Sophoricoside contains two aromatic rings. For form A, the C=O stretching vibrations appear at 1655 and 1618 cm^−1^, and the C=C stretching vibrations appear at 1574 and 1506 cm^−1^. For form B, the C=O stretching vibrations appear at 1654 and 1622 cm^−1^, and the C=C stretching vibrations appear at 1574, 1519, and 1508 cm^−1^. For form C, the C=O stretching vibrations appear at 1659 cm^−1^, and the C=C stretching vibrations appear at 1610, 1596, 1576 and 1509 cm^−1^. Form B contains 0.5 DMSO. The S=O stretching vibration of pure liquid DMSO occurs at 1050 cm^−1^ [26]. However, for form B, the S=O stretching vibration appears at about 1009 cm^−1^ because of strong hydrogen-bonding interactions between the solvent and host molecule, which shift the bands toward lower frequencies. Forms D and A contain the same elemental composition, but the molecular arrangement of form D is disordered, so the FT-IR spectrum of form D has a small number of peaks and a broad peak shape. The main vibrational data with assignments for the four sophoricoside forms are shown in electronic supplementary material, table S2.

### 3.5. Stability and transformation

The tendency for stability and transformation among the solid-state forms were examined by high-temperature (60°C) experiments, high-humidity (90% ± 5%, 25°C) experiments and strong light (4500 lx ± 500 lx) experiments. Form A could remain stable at high-temperature, high-humidity and strong light experiments for 10 days. But forms B and C were unstable at the high-temperature (60°C) test; they easily desolvated and transformed to form A in 5 days. Form D was unstable at high humidity (90% ± 5%, 25°C) test; it easily transformed to form A in 5 days (electronic supplementary material, figure S2).

### 3.6. Solubility

**Figure 8.**
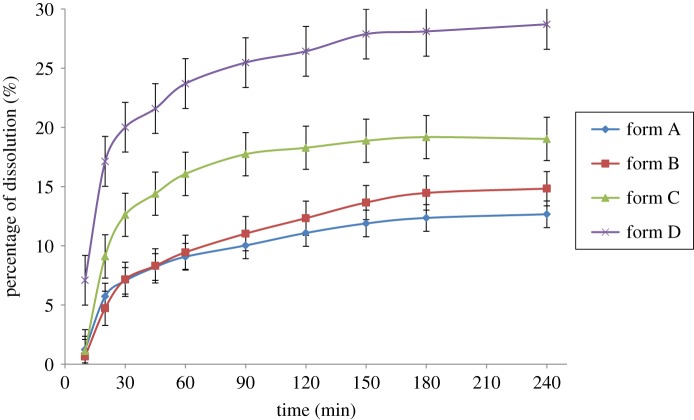
Solution profiles of four solid-state forms of sophoricoside in pure water.

The time–solubility profiles of four solid-state forms of sophoricoside in pure water at 310 K are presented in figure 8. The solubility of the amorphous phase and solvatomorphism are greater than that of solvent-free form. Amorphous phase exhibits the greatest solubility, as high as 28.7%, similar to the solubility reported—amorphous state was used to improve the solubility of poorly soluble drugs. From the SXRD results, the crystal density value of form A was higher than those of forms B and C, so the solubility of form A is the lowest. Water molecules permeate into the structure of form C and cause form C to disintegrate in pure water; simultaneously form C arranges loosely and has lower density, which may be responsible for its higher solubility relative to form B.

Sophoricoside is a poorly water-soluble compound. Amorphous phase and solvatomorphism can obviously improve the solubility of the compound, especially amorphous phase.

## 4. Conclusion

Sophoricoside is an important compound; it has various biological activities and potential therapeutic effects. Four solid-state forms of sophoricoside were identified and prepared by physical or chemical methods. This is the first time that these four forms have been reported. The solid-state properties of the four forms were investigated using various analytical techniques. SXRD showed that differences among the molecular conformations, solvent incorporation and the spatial arrangement led to the formation of sophoricoside solvatomorphism. In addition, simulated PXRD patterns were obtained from SXRD data for forms A–C and provided a standard for determining the crystalline purity. TGA and DSC were used for characterization of the solid-state forms of sophoricoside, to identify the type and amount of solvent present. FT-IR spectroscopy was also used for identification because of the obvious differences at the regions of hydrogen bonds, functional groups and fingerprint. In addition to the solid-state properties, stability and solubility of the four solid-state forms were studied. The stability results showed that solvatomorphism lost the crystallization solvents and converted to form A at high temperatures; form D transformed to form A at high humidity (90% ± 5%, 25°C). The solubility results showed that amorphous phase and solvatomorphism of sophoricoside can obviously improve the solubility, especially the percentage of dissolution of the form D was approximately three times that of form A. If sophoricoside is developed as a medicine, perhaps form D is a good choice.

## Supplementary Material

Table S1. d-Spacings (Å), 2θ values (°), and relative intensities (%) of ten most intense peaks in simulated XRPD patterns of sophoricoside forms;Table S2. Data for main vibrational peaks (cm-1), with assignments, for sophoricoside forms ;Fig. S1.Forms B, C and D of sophoricoside transform to form A at more than 100 °C..;Fig. S2. Forms B, C and D of sophoricoside transform to form A at influencing factor test.

## Data accessibility

PXRD, IR, DSC, TG data files are available at the Dryad Digital Repository: http://dx.doi.org/10.5061/dryad.5qd036v [27].

SXRD data files: The Cambridge Crystallographic Data Centre (CCDC): reference numbers 1812874–1812876. (https://www.ccdc.cam.ac.uk/MyStructures/).
