# Supplementary material for: New crystal forms and amorphous phase of sophoricoside: X-ray structures and characterization
Source: R Soc Open Sci. 2019 Jan 23;6(1):181905. doi: 10.1098/rsos.181905 (PMC6366211; doi:10.1098/rsos.181905)
Supplement: Table S1. d-Spacings (Å), 2θ values (°), and relative intensities (%) of ten most intense peaks in simulated XRPD patterns of sophoricoside forms;Table S2. Data for main vibrational peaks (cm-1), with assignments, for sophoricoside forms ;Fig. S1.Forms B, C and D of sophoricoside transform to form A [file rsos181905supp1.docx]

Table S1. *d*-Spacings (Å), 2*θ* values (°), and relative intensities (%) of ten most intense peaks in simulated XRPD patterns of sophoricoside forms

| No. | Form A | | | | Form B | | | Form C | | | Form D | | |
| --- | --- | --- | --- | --- | --- | --- | --- | --- | --- | --- | --- | --- | --- |
|  | *d* | 2*θ* | *I*/*I*_0_ | *d* | | 2*θ* | *I*/*I*_0_ | *d* | 2*θ* | *I*/*I*_0_ | *d* | 2*θ* | *I*/*I*_0_ |
| 1 | 3.86 | 23.06 | 100 | 3.78 | | 23.52 | 100.0 | 11.92 | 7.44 | 100.0 | 3.92 | 22.66 | 100.0 |
| 2 | 6.12 | 14.46 | 87.7 | 3.23 | | 27.64 | 40.8 | 23.56 | 3.76 | 76.0 | 6.14 | 14.42 | 84.4 |
| 3 | 3.26 | 27.32 | 63.5 | 4.85 | | 18.28 | 39.9 | 4.16 | 21.32 | 56.7 | / | / | / |
| 4 | 4.32 | 20.56 | 37.1 | 6.40 | | 13.92 | 35.1 | 6.63 | 13.34 | 56.5 | / | / | / |
| 5 | 4.81 | 18.46 | 30.7 | 5.68 | | 15.62 | 28.1 | 5.19 | 17.08 | 55.6 | / | / | / |
| 6 | 3.01 | 29.64 | 27.8 | 4.29 | | 20.70 | 26.2 | 4.57 | 19.64 | 52.3 | / | / | / |
| 7 | 3.17 | 28.10 | 21.2 | 3.72 | | 23.86 | 24.6 | 3.90 | 22.82 | 34.9 | / | / | / |
| 8 | 4.68 | 18.94 | 15.0 | 6.19 | | 14.40 | 22.8 | 18.55 | 4.76 | 34.6 | / | / | / |
| 9 | 3.78 | 23.50 | 10.8 | 4.66 | | 19.10 | 19.7 | 6.82 | 12.96 | 26.9 | / | / | / |
| 10 | 5.21 | 17.00 | 10.6 | 4.70 | | 22.02 | 18.8 | 3.70 | 24.02 | 21.6 | / | / | / |

Table S2. Data for main vibrational peaks (cm^-1^), with assignments, for sophoricoside forms

| Vibrational data | | | | Vibrational assignment |
| --- | --- | --- | --- | --- |
| Form A | Form B | Form C | Form D |  |
| 3674, 3583, 3243 | 3675, 3447, 3198 | 3463, 3192 | 3657,3511 | H_2_O, O–H stretching vibrations |
| 2976, 2951, 2901 | 2988, 2932, 2901, 2884 | 2940, 2875 | 2957, 2933 | CH_3_, CH_2_, C–H stretching vibrations |
| 1655, 1618 | 1654, 1622 | 1659 | 1655 | C=O stretching vibrations |
| 1574, 1506 | 1574, 1519, 1508 | 1610, 1596, 1576, 1509 | 1574, 1510 | C=C stretching vibrations |
| 1473, 1367 | 1406, 1366 | 1482, 1442, 1395, 1337 | 1450, 1411, 1365 | C–H symmetrical and asymmetrical deformation vibrations |
| 1298, 1280, 1254, 1228 | 1297, 1278, 1250, 1230, 1204 | 1284, 1233, 1201 | 1286, 1231 | =C–O stretching vibrations |
| 1177, 1072, 1047 | 1180, 1074, 1049, 1009 | 1181, 1150, 1115, 1076, 1045, 1031 | 1179, 1129 | =C–O stretching vibrations |
| - | 1009 | - | - | S=O stretching vibration |
| 897, 825 | 879, 824 | 877, 834 | 882, 826 | =CH_2_ wagging vibrations |


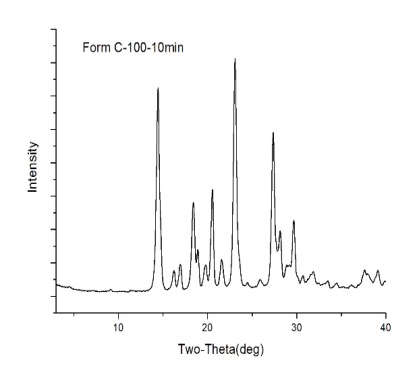

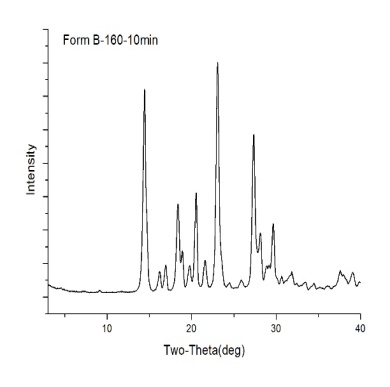

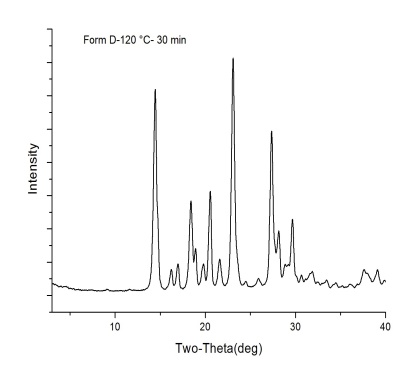


Fig. S1.Forms B, C and D of sophoricoside transform to form A at more than 100 °C..

Form B-160 °C-10 min: form B is converted to form A at 160 °C after 10 min.

Form C-100 °C- 10 min: form C is converted to form A at 100 °C after 10 min.

Form D-120 °C- 30 min: form D is converted to form A at 120 °C after 30 min.


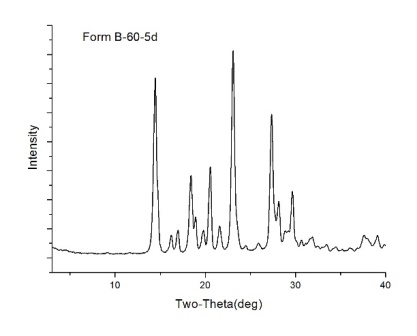

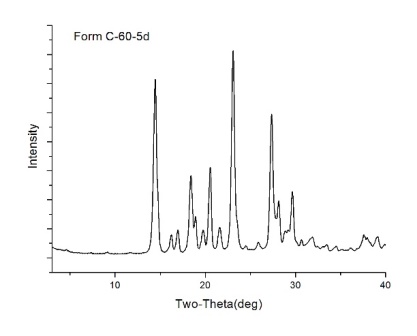

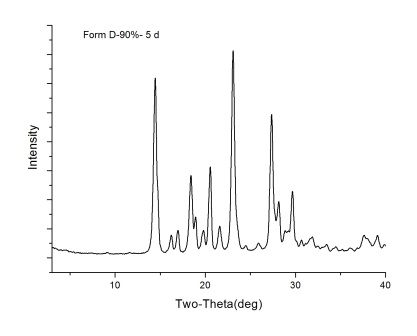


Fig. S2. Forms B, C and D of sophoricoside transform to form A at influencing factor test.

Form B-60 °C- 5 d: form B is converted to form A at high temperature (60 °C) after 5 d.

Form C-60 °C- 5 d: form C is converted to form A at high temperature (60 °C) after 5 d.

Form D-90%- 5 d: form D is converted to form A at high humidity (90% ± 5%, 25 °C) after 5 d.
